# Efficient derivation of dopaminergic neurons from SOX1^−^ floor plate cells under defined culture conditions

**DOI:** 10.1186/s12929-016-0251-6

**Published:** 2016-03-08

**Authors:** Mingming Li, Yu Zou, Qiqi Lu, Ning Tang, Alexis Heng, Intekhab Islam, Huei Jinn Tong, Gavin S. Dawe, Tong Cao

**Affiliations:** Faculty of Dentistry, National University of Singapore, Kent Ridge, Singapore; Department of Pharmacology, Yong Loo Lin School of Medicine, The National University of Singapore, Kent Ridge, Singapore; Neurobiology and Ageing Programme, Life Sciences Institute of the National University of Singapore, Kent Ridge, Singapore; Singapore Institute for Neurotechnology (SINAPSE), The National University of Singapore, Kent Ridge, Singapore; Faculty of Dentistry, The University of Hong Kong, Pokfulam, Hong Kong; Tissue Engineering Program, Life Sciences Institute of the National University of Singapore, Kent Ridge, Singapore; National University of Singapore Graduate School for Integrative Sciences and Engineering (NGS), Kent Ridge, Singapore

**Keywords:** Human embryonic stem cells, Neural conversion, Floor plate, LDN193189, SB431541, PD173074, Dopaminergic neurons

## Abstract

**Background:**

Parkinson’s disease (PD) is a severe neurodegenerative disease associated with loss of dopaminergic neurons. Derivation of dopaminergic neurons from human embryonic stem cells (hESCs) could provide new therapeutic options for PD therapy. Dopaminergic neurons are derived from SOX^−^ floor plate (FP) cells during embryonic development in many species and in human cell culture in vitro. Early treatment with sonic hedgehog (Shh) has been reported to efficiently convert hESCs into FP lineages.

**Methods:**

In this study, we attempted to utilize a Shh-free approach in deriving SOX1^−^ FP cells from hESCs in vitro. Neuroectoderm conversion from hESCs was achieved with dual inhibition of the BMP4 (LDN193189) and TGF-β signaling pathways (SB431542) for 24 h under defined culture conditions.

**Results:**

Following a further 5 days of treatment with LDN193189 or LDN193189 + SB431542, SOX1^−^ FP cells constituted 70–80 % of the entire cell population. Upon treatment with Shh and FGF8, the SOX1^−^ FP cells were efficiently converted to functional Nurr1^+^ and TH^+^ dopaminergic cells (patterning), which constituted more than 98 % of the entire cell population. However, when the same growth factors were applied to SOX1^+^ cells, only less than 4 % of the cells became Nurr1^+^, indicating that patterning was effective only if SOX1 expression was down-regulated. After transplanting the Nurr1^+^ and TH^+^ cells into a hemiparkinsonian rat model, significant improvements were observed in amphetamine induced ipslateral rotations, apomorphine induced contra-lateral rotations and Rota rod motor tests over a duration of 8 weeks.

**Conclusions:**

Our findings thus provide a convenient approach to FP development and functional dopaminergic neuron derivation.

**Electronic supplementary material:**

The online version of this article (doi:10.1186/s12929-016-0251-6) contains supplementary material, which is available to authorized users.

## Background

Parkinson’s disease (PD) is the second most common central nervous system neurodegenerative disease and the most common sub-cortical neurodegenerative disease [1]. The prevalence of PD is generally estimated at 0.3 % of the entire population, 1 % of the population over 60, and 4 % of those over 80 [2]. PD is caused by loss of dopaminergic neurons from the substantia nigra and disease severity is correlated to the percentage of dopaminergic neuronal loss [3]. PD in most people is idiopathic but environmental or genetic factors may contribute to the onset of dopaminergic neuron loss in certain cases. Currently, there is no proven preventative therapy or cure for this disease.

PD is typically managed pharmacologically with dopaminergic agonist replacement or by increasing production of dopamine in surviving dopaminergic neurons through administration of the dopamine precursor, L-3,4-dihydroxyphenylalanine (L-DOPA). In certain cases, deep brain stimulation and fetal tissue transplantation have been utilized in the clinical management of PD [4]. The transplantation of donor fetal tissue is however associated with concerns regarding supply of sufficient quantities of tissue, inter-batch variability, safety and ethics. Human embryonic stem cells (hESCs) hold great promise in regenerative medicine. There is potential for application in PD therapy as hESCs have been differentiated into functional dopaminergic neurons with various protocols [5–7]. Of particular interest is the study by Kriks et al. that reported a floor plate (FP)-based strategy for the derivation of human DA neurons within 25 days [7], based on the earlier FP induction protocol developed by Fasano et al. that involved early sonic hedgehog (Shh) exposure [8].

As an alternative to Shh-induced FP induction, Teillet et al. showed that the FP develops in a cell-autonomous manner in the absence of a notochord, providing evidence that the lineage fate of some FP cells derived from the chordoneural hinge is predetermined [9]. In this study, we employed a Shh-free approach in deriving FP cells with BMP and TGF-β dual inhibition. To determine the optimal timing of FP induction for our in vitro system, we utilized the dynamics of SOX1 expression as an indicator. Studies have shown that SOX1 is restricted to neural folds at the 2nd somite stage and restricted to the neural tube at the 10th-12th somite stages. At the 20th somite stage, SOX1 is down-regulated in the FP of the neural tube. Specifically, cells occupying the lateral regions of the FP express SOX1, whereas cells occupying the medial region do not [10, 11]. However, little is known about how SOX1 is associated with FP cells derived in situ within the developing human embryo or equivalent in vitro model systems.

In this study, we report efficient induction of SOX1^−^ FP cells for the derivation of dopaminergic neurons from hESCs. Dissociation of hESC into a single-cell suspension was carried out, followed by treatment (for 24 h) with a BMP4 inhibitor, LDN193189, and a TGF-β inhibitor, SB431542, (dual restriction) for neuroectoderm conversion under defined culture conditions. The cells were then subsequently cultured in the presence of LDN193189 and SB431542 (dual inhibition) for 5 days to achieve a SOX1^−^ medial FP cell population. In addition, 5 days of treatment with FGF2 or transient treatment with its inhibitor PD173074 was investigated in our platform (patterning). We found that when the cells are SOX1^+^, treatment with Shh and FGF8 only yielded less than 4 % of Nurr1^+^ cells. It was only after SOX1 expression was down-regulated, that Shh and FGF8 treatment yielded more than 90 % Nurr1^+^ cells.

## Methods

### Cell lines, culture media and supplements, labware consumables and reagents

Undifferentiated hESC of the H1 and H9 line were purchased from Wicell Inc. (Madison, WI, USA). Unless otherwise stated, all culture media and supplements including serum and trypsin were purchased from Gibco BRL Inc. (Grand Island, NY, USA); all cell culture plastic labware consumables were purchased from BD Bioscience Inc. (Franklin Lakes, NJ, USA); and all reagents including growth factors were purchased from Sigma-Aldrich Inc. (St Louis, MO, USA). Cell cultures were carried out at 37 °C within a humidified 5 % CO_2_ incubator (Heraeus GmbH, Hanau, Germany).

### Cell culture and GFP labeling

H1 and H9 hESCs were continuously co-cultured under Mytomycin-C inactivated Mouse Embryonic Fibroblast (MEF) feeders, as previously described [12]. The hESCs culture medium is composed of Dulbecco’s modified Eagle’s medium (DMEM) F-12 supplemented with 20 % (v/v) knock-out serum replacement (KSR), 1 mM L-glutamine, 0.1 mM β-mercaptoethanol, 1 % (v/v) non-essential amino acid and 4 ng/ml recombinant basic fibroblast growth factor (rFGF2). Culture media were changed every day and cells were sub-cultured at 5–6 days intervals. For labeling, hESCs colonies were treated with 2 mg/ml collagenase IV for 20 min to allow the colonies to detach from the MEF feeders. The floating colonies were then collected and dissociated with trypsin/enzyme free cell dissociation buffer mixture (3:7) in the presence of Y-27632. A plasmid vector carrying the GFP gene was incubated with the hESCs for 24 h in Ultra-low Attachment plates. Transfected cells were then seeded again on MEF feeder layers for expansion from single cells to colonies. Green-fluorescent colonies were manually selected under fluorescent microscopy for expansion and differentiation.

### Induction of FP neurons and patterning

hESCs were detached with 2 mg/ml collagenase type IV for 20mins and floating colonies were collected. The pellets were then dissociated with trypsin/enzyme free cell dissociation buffer mixture (3:7) in the presence of Y-27632. Dissociated cells were then re-suspended into DMEM/F-12 medium supplemented with 20 % (v/v) KSR containing 2nM LDN193189 for single restriction or 2nM LDN193189 and 10 μM SB431542 for dual restriction for 24 h on fibronectin-coated plates. For FP induction, cells were cultured in neural basal medium (NB, Life technologies Inc. Grand Island, NY, USA) supplemented with 1 mM L-glutamine, 1 % (v/v) non-essential amino acid, 1 % (v/v) N2 supplement with or without 0.5nM LDN193189 or 10 μM SB431542 for another 5 days. The FGF2 inhibitor, PD173074 (1 nM), was added immediately after dual restriction for 24 h only for lateral FP cell induction. For patterning, cells were subsequently cultured in NB supplemented with 1 % (v/v) NEAA, 1 mM L-glutamine, 1 % (v/v) B27 supplement, 200 ng/ml Shh, and 100 ng/ml FGF8. Shh and FGF8 were then withdrawn and replaced with 20 ng/ml brain derived neurotrophic factor (BDNF) and 20 ng/ml glial cell derived neurotrophic factor (GDNF), 1 μM Transforming growth factor beta-3, 200 μM ascorbic acid and 1 mM Dibutyryl cyclic adenosine monophosphate (dcAMP), to maintain the differentiated cells [13].

### Gene expression analysis by polymerase chain reaction

mRNA was extracted from samples using Qiagen RNeasy® Mini Kits (Qiagen Inc., Hilden, Germany). Following the manufacture’s instructions, total mRNA was immediately quantified by nanodrop and 2 μl of each mRNA sample was loaded to a 1.5 % agarose gel for integrity checking. 500 ng of mRNA was reverse transcribed to cDNA using the Biorad iScript™ cDNA synthesis kits (Biorad Inc., Hercules, CA, USA). Briefly, 40 μl of cDNA was synthesized with programmed temperature changes of 5 min at 25 °C, then 30 min at 42 °C and followed by 85 °C for 5 min to heat-inactivate the reverse transcriptase. For qRT-PCR, 20 μl volume of reaction mix was utilized (including 10 μl Fast SYBR® Green Master Mix (2X) (Life technologies Inc. Grand Island, NY, USA), 5nM forward and reverse primers 0.4 μl each, and RNAase free water). cDNA was denatured at 95 °C for 10mins, followed by 40 cycles of denaturation at 95 °C for 10 s and 60 °C for 1 min. All runs were performed on a StepOnePlus™ Real-Time PCR System (Life technologies Inc. Grand Island, NY, USA) with GAPDH utilized as the loading control. Fold differences were calculated relative to control samples. All primers utilized are listed in Additional file 1: Table S1. For conventional PCR, a volume of 20 μl was utilized (including 10 μl Fermentas Master Mix (2X) (Thermo-Scientific Inc., Waltham, MA, USA), 25nM forward and reverse primers 0.4 μl each, and RNAase free water). The PCR product was loaded on a 1.5 % agarose gel and imaged with a Bio-rad Gel-Doc system (Biorad Inc., Hercules, CA, USA).

### Flow cytometry

Cells were detached by trypsinization for 5 min at 37 °C. After fixation in 4 % (w/v) PFA for 10 min at room temperature, the cells were immediately chilled on ice for 1 min, followed by 10mins permeabilization in 0.2 % Triton X-100 in 1X PBS. The cells were then incubated with the appropriate primary antibodies for 4 h at room temperature followed by incubation with corresponding secondary antibodies for 1 h, prior to analysis with a CyAn™ Flow Cytometer (Beckman-Coulter Inc., Indianapolis, IN, USA). All secondary antibodies used were tested for cross reactivity and non-specific immunoreactivity. Approximately, 10,000 cells were gated for analyzing each marker.

### Immunocytochemistry

Cells/brain slices were washed with 1X phosphate buffered saline (PBS) three times and fixed with 4 % (w/v) Paraformaldehyde (PFA) for 10mins at room temperature. Following 10mins permeabilization with 0.2 % Triton X-100 in PBS, the cells were blocked for 1 h with 5 % (v/v) goat serum and 2 % (w/v) bovine serum albumin (BSA). Diluted primary antibodies were then added to the culture and incubated at 4 °C overnight. After 4 washes with 1X PBS, diluted secondary antibodies against the specific primary antibodies were added and incubated in the dark at room temperature for 1 h. All secondary antibodies used were tested for cross reactivity and nonspecific immunoreactivity. The nuclear stain DAPI was added 10mins prior to the final wash. Images were captured under fluorescence microscopy. The antibodies used are listed in Additional file 2: Table S2.

### Live imaging analysis for neurite outgrowth

Differentiating dopaminergic cells from monolayer culture of FP origin were analyzed for neurite outgrowth. Cells generated by a neurosphere protocol were imaged together for comparison [14]. Differentiating dopaminergic neurons in 24-well plates were continuously cultured in a Cell IQ Imagen system (Chip-Man Technologies INC., Tampere, Finland) for 5 days in a Cell IQ incubator with 5 % CO_2_ mixed with air at 37 °C. 2 × 2 grid images were sampled continuously every 15 min on selected colonies collected over a period of 5 days with 10X magnification using the Cell IQ Imagen system. Superimposed images were then analyzed using the Neurite Finder™ module of the Cell IQ Analyser.

### In vitro patch clamp

Differentiated dopaminergic cells were immersed in an external solution of 125 mM NaCl, 3 mM KCl, 1.25 mM NaH_2_PO_4_, 1.5 mM MgCl_2_, 2 mM CaCl_2_, 24 mM NaHCO_3_, and 10 mM D-Glucose, with pH adjusted to 7.4. Whole-cell voltage clamping was conducted with the membrane potential clamped at -60 mV as the resting potential of the differentiated neurons. The currents were recorded with borosilicate pipettes with resistances of 2–6MΩ, which were pulled from borosilicate glass capillaries with a Flaming Brown micropipette puller. The pipettes were filled with an internal solution with the same composition as the external solution. Transplanted neurons were clamped using a MultiClamp 700A amplifier in conjunction with a Digidata 1322A interface (Molecular Devices Inc., Sunnyvale, CA, USA). Currents were recorded and analyzed using the pCLAMP software (Molecular Devices Inc., Sunnyvale, CA, USA).

### Dopamine secretion assay

Dopamine secretion into the culture media was analyzed using a Dopamine ELISA Kit (GenWay Biotech Inc., San Diego, CA, USA) following the manufacture’s instructions with slight modifications. For amantadine treated samples, 5 or 10 μM of amantadine was added to the culture media. Culture media were collected every 3 days after induction for quantification of dopamine secretion. Briefly, dopamine from culture media was first extracted with 12 well pre-coated extraction plates. Following a few washes, bound dopamine was extracted by 200 μl of release buffer. Enzyme solution, 100 μl of the extracted samples and dopamine anti-serum were added to pre-coated 96 well plates and incubated for 2 h at room temperature. Enzyme conjugate and PNPP substrate solution were added sequentially with washes in-between. The reaction was stopped by addition of PNPP stop solution and absorbance of samples were read at 405 nm using Infinite™ 200 plate readers (Tecan Inc., Männedorf, Switzerland). The amounts of secreted dopamine were calculated based on a standard curve plotted with a series of known dopamine dilutions supplied by the manufacturer.

### Transplantation into Hemi-Parkinsonian rats, behavioral analysis, patch clamp and histological staining

Sprague Dawley rats were purchased from the animal holding unit (AHU) of the National University of Singapore, and were housed in biological safety level 2 pathogen-free facilities with free access to food and water under a 12 h light/dark cycle, and with standard room temperature of 23 ± 1 °C. Ethical approval for all animal experiments were obtained from the Institutional Animal Care and Use Committee (IACUC) of the National University of Singapore. All animal studies were in compliance with the “Animal Research: Reporting In Vivo Experiments” (ARRIVE) guidelines. Animals (250 g) were anaesthetized and immobilized with a stereotactic instrument. 20 μg of 6-OHDA was stereotaxically injected at the bregma (AP +1.00 mm, ML +3.00 mm, DV +4.5 mm), at a concentration of 5 μg/μl with the aid of the Angle Two Software for unilateral lesion. Only animals with a mean contralateral rotation score of 7 or more body turns per minute at 3 weeks post-lesion were included in the study. Initially, lesions were created on 25 rats. However by 3 weeks post-lesion, only 12 rats that met the criteria of >7 rounds of contra-literal rotation induced by 0.5 mg/lg apomorphine were utilized for cell transplantation. At 12 days post-lesion, dopaminergic cells (250,000 cells/4 μl) were grafted into the same lesion site. 0.5 mg/kg of apomorphine or 0.25 mg/kg of amphetamine were injected subcutaneously or intraperitoneally and their contralateral and ipslateral rotation behavior was monitored over a 45 min period using an EthoVsion XT system (Noldus Inc., Wageningen, Netherlands). Rotation scores were expressed as full body turns per minute. To measure balance and coordination, animals were placed on a horizontally rotating rod that accelerated from 2rmp to 40 rpm within 2 min. The latency of fall off from the rod was recorded for 6 trials with 10 min rest between each trial.

Eight weeks after transplantation of GFP-labeled cells, brain samples were sectioned at 200 μm with a vibratome in 10 % (v/v) glycerol in PBS. Whole-cell voltage clamping was conducted with the membrane potential clamped at -60 mV as the resting potential of the differentiated neurons. The currents were recorded with borosilicate pipettes with resistances of 2–6MΩ, filled with an internal solution containing (in): 140 mM K Gluconate, 20 mM HEPES, 2 mM MgCl_2_, 4 mM K_2_ATP, 2 mM NaCl, 1 mM EGTA, and 2 mM NaCl, with pH and osmolarity of the solutions being adjusted to 7.2 and 290 mOsm respectively. The external solution was composed of 150 mM NaCl, 5 mM KCl, 2 mM CaCl_2_, 10 mM HEPES, 1 mM MgCl_2_, 10 mM glucose with the pH and osmolarity of the solution being adjusted to 7.2 and 310 mOsm respectively. Transplanted neurons were clamped using a MultiClamp 700A amplifier in conjunction with a Digidata 1322A interface (Molecular Devices Inc., Sunnyvale, CA, USA). Currents were recorded and analyzed using the pCLAMP software (Molecular Devices Inc., Sunnyvale, CA, USA).

10 μm brain slices sectioned with a vibrotome were also stained with haematoxylin and eosin to determine whether there is any teratoma formation by the transplanted cells. Briefly, sections were fixed in 4 % (w/v) paraformaldehyde and washed in PBS for 4 times followed by 1 min staining with haematoxylin. Samples were then rinsed in distilled deionised water until no dye can be extracted from the samples, followed with eosin staining for 2mins. Samples were then washed, dehydrated and mounted on glass slides.

### Statistical analysis

Numerical data of experimental and control groups were analyzed with one-way Anova Bonferroni post-hoc multi-group comparison, utilizing the SPSS software. A value of *P* < 0.05 was considered statistically significant.

## Results

### LDN193189 treatment restricts hESC lineage fate to neuroectoderm

**Fig. 1:**
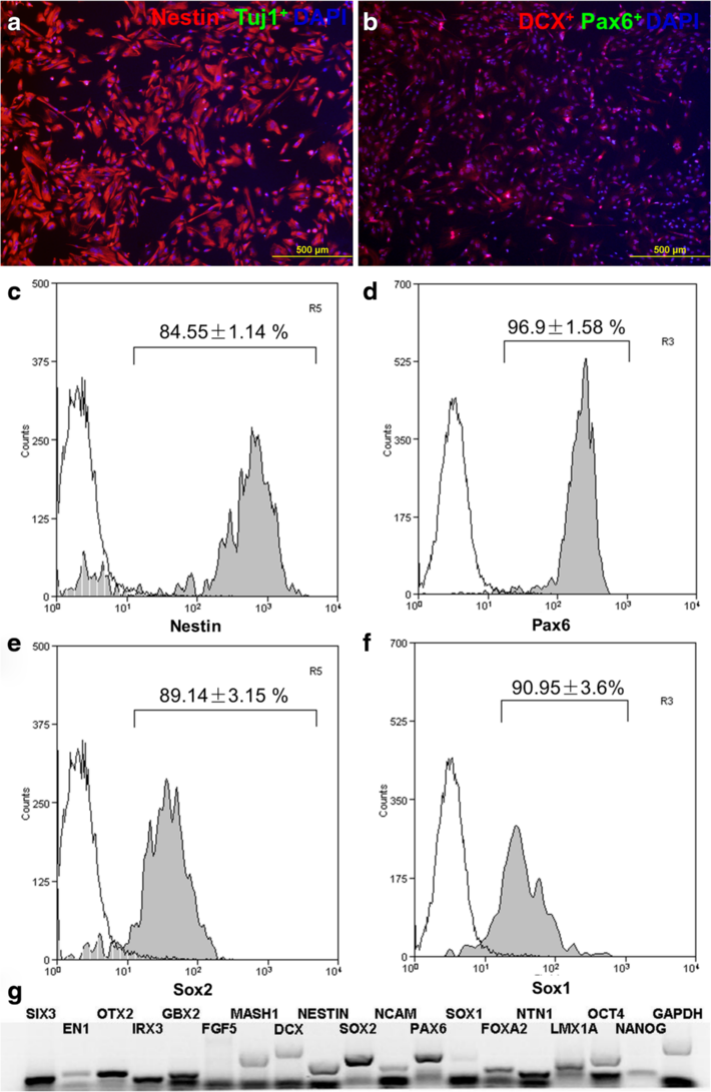
Under defined culture condition, when cells were treated with LDN193189 for 24 h, cells remained Nestin^+^ (**a**, *red*) and DCX^+^ (**b**, *red*) after 7 days of culture in EB medium. After lineage restriction and 5 days continuous culture in Neural basal medium supplemented with N2, 84.55 ± 1.14 % of the cells were Nestin^+^ (**c**), 96.9 ± 1.58 % of the cells were Pax6^+^ (**d**), 89.14 ± 3.15 % of the cells were SOX2^+^ (**e**) and 90.95 ± 3.6 % of the cells were SOX1^+^ (**f**). Neural plate (NP) markers IRX3, SIX3 and OTX2, FP markers FOXA2 and NTN1, and neuroepithelial cell markers SOX2 and PAX6 are all highly expressed after 5 days (**g**). Scale bars: A, B, 500 μm

During gastrulation in embryo development, bone morphogenetic protein 4 (BMP4) is expressed in the embryo at the time of ectodermal fate determination. The nervous system forms when BMP4 signaling is inhibited [15]. Treatment with the BMP4 inhibitor LDN193189 results in effective induction of neural cells from hESCs [16]. Studies in mouse embryonic stem cells have also shown that the cells assume a primitive neural stem cell fate in the absence of extrinsic influences within in vitro culture [17]. We conducted a preliminary screening study whereby neural marker expression of hESC was analyzed after treatment with increasing dosages (1, 2 and 5nM) of LDN193189 for varying durations of 24 h, 48 h and 72 h. Untreated hESC was utilized as the control. The results (Additional file 3: Figure S1) showed that treatment with 2nM LDN193189 for 24 h yielded the highest neural marker expression on average, as compared to other dosages. Although there was marked increase in SOX1 expression after 48 h treatment with 2nM LDN193189, low cell viability was observed. Hence, we initially treated hESC with a dosage of 2 μM LDN193189 for 24 h. After LDN193189 treatment for 24 h, the cells remain restricted to Nestin^+^ (Fig. 1a) and DCX^+^ (Fig. 1b) neuroectodermal fate, even after 7 days of culture in ES medium without FGF2. After LDN193189 treatment and 5 days of continuous culture in N2 supplemented culture medium, the four selected neuroectoderm markers were all up-regulated, with 84.55 ± 1.14 % of the cells expressing Nestin^+^ (Fig. 1c), 96.9 ± 1.58 % of the cells expressing Pax6^+^ (Fig. 1d), 89.14 ± 3.15 % of the cells expressing SOX2^+^ (Fig. 1e) and 90.95 ± 3.6 % of the cells expressing SOX1^+^ (Fig. 1f). In addition, neural plate (NP) markers: IRX3, SIX3, and OTX2; FP markers: FOXA2, and NTN1, and general neuroepithelial cell markers: SOX2 and PAX6 are all highly expressed after 5 days of culture (Fig. 1g).

### Dual inhibition produces medial FP cells

**Fig. 2:**
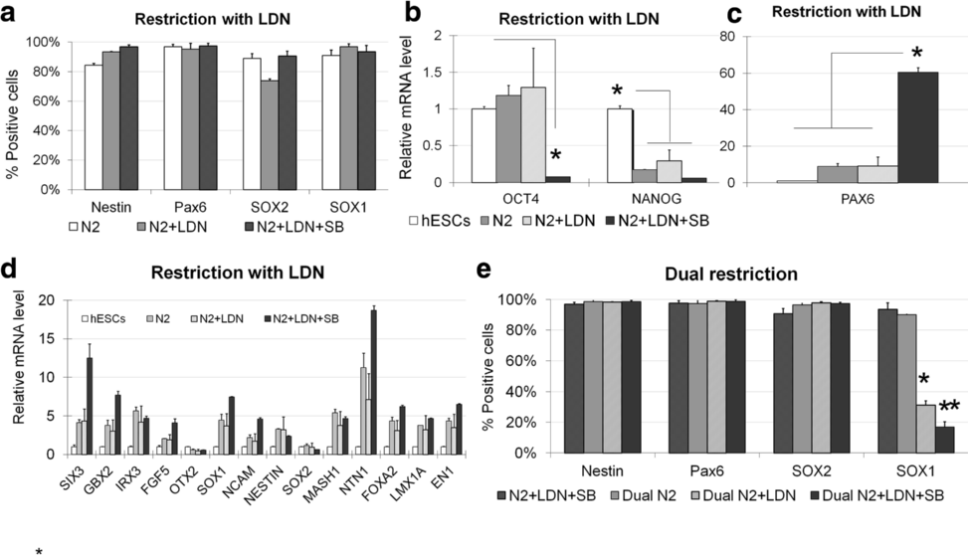
**a** Following LDN193189 restriction for 24 h, when cells were cultured under N2, N2 and LDN193189, or dual inhibition medium, the percentage of Nestin^+^ cells increased from 84.55 ± 1.14 % to 96.84 ± 1.33 %. By contrast, no obvious changes were observed for Pax6^+^, SOX1^+^ and SOX2^+^ cells. **b** Compared with hESCs, OCT4 expression was downregulated significantly only after LDN193189 restriction followed by dual inhibition. NANOG was downregulated significantly after LDN193189 restriction followed by N2, N2 and LDN193189 or dual inhibition medium. **c** After LDN193189 restriction, when cells were cultured in dual inhibition medium for 5 days, Pax6 expression increased significantly compared with undifferentiated hESCs, culture in N2 supplemented medium or LDN193189 inhibition for 5 days. **d** After lineage restriction with LDN193189 treatment, 5 days of dual inhibition resulted in significantly higher levels of neural gene expression comparing with the other treatment groups. **e** After dual lineage restriction, 5 days of continuous LDN1931898 inhibition or dual inhibition resulted in a significantly lower percentage of SOX1^+^ cells. *n* = 3 for each group

To further optimize the differentiation condition, 0.5nM LDN193189 was continuously added to the induction medium, but no differences in gene expression levels of neural markers were observed. However, the proportion of Nestin^+^ cells increased slightly to 93.38 ± 0.22 %, with no significant changes in the expression of the other 3 markers: Pax6, SOX2 and SOX1 (Fig. 2a). Similar to BMP4 inhibition, the TGF-β inhibitor SB431542, which also targets downstream SMAD signaling, has also been reported to have potent effects on neural induction of hESCs [18, 19]. Additionally, dual SMAD inhibition has been proven to efficiently convert hESCs to the neural lineage [20]. When both LDN193189 and SB431542 were added to the induction medium (dual inhibition), Pluripotency marker OCT4 and NANOG were downregulated to an undetectable level (Fig. 2b). NP markers SOX1, SIX3, and FGF5; FP markers FOXA2 and NTN1, were all up-regulated (Fig. 2d). Specifically, PAX6 was up-regulated by 60-fold (Fig. 2c) and the proportion of Nestin^+^ cells significantly increased again up to 96.84 ± 1.33 % (Fig. 2a). Interestingly, when SB431542 was applied earlier during the first 24 h of culture, dual restriction alone followed by induction medium had the same effect as initial treatment with LDN193189 (24 h) followed by dual inhibition (5 days) on cellular expression of Nestin, Pax6, SOX2 and SOX1 (Fig. 2e). Continuous inhibition with LDN193189 for 5 days after dual restriction with both LDN193189 and SB431542 for the first 24 h, resulted in a reduction of the proportion of SOX1^+^ cells to 31.16 ± 2.82 %; whereas continuous dual restriction with both LDN193189 and SB431542 for the first 24 h followed by dual inhibition for 5 days significantly reduced the SOX1^+^ population to 16.88 ± 3.48 % (Fig. 2e).

### FGF2 inhibits neural induction and inhibiting FGF2 signaling sustains NP state

**Fig. 3:**
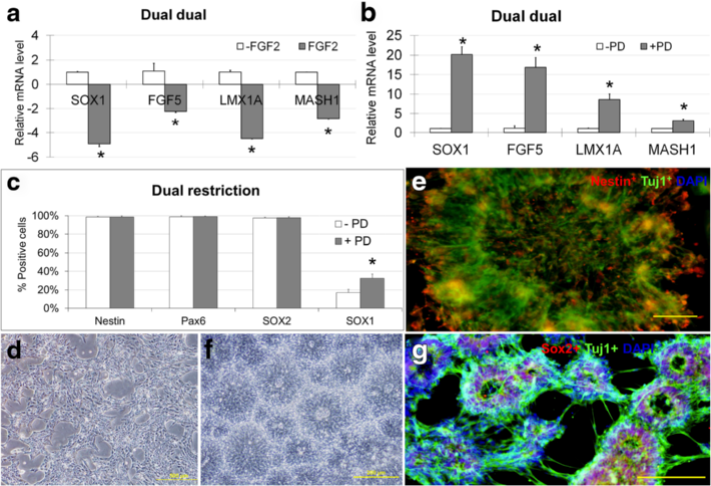
**a** Under dual restriction and dual inhibition, when FGF2 were supplemented to the culture medium during dual inhibition, SOX1, FGF5, LMX1A and MASH1 were all significantly down-regulated. **b** Under dual restriction and dual inhibition, when PD173074 were added to the culture medium on day 1, there was significant up-regulation of SOX1, FGF5, LMX1A and MASH1 expression. **c** Under dual restriction and dual inhibition, a significantly higher percentage of SOX1^+^ cells was observed for cells treated with PD173074 on day 1 only and harvested on day 5, as compared to the absence of PD173074 treatment. **d** Morphologically, cells formed networked structures on day 5 when switched to differentiation medium from restriction medium. **e** After 5 days of culture, Nestin^+^ (*Red*) and Tuj1^+^ (*Green*) were found co-localized within the colonies formed. **f** When sub-cultured, columnar neural rosettes were observed on day 10. **g** When sub-cultured, columnar neural rosettes were SOX2^+^ (*Red*) and Tuj1^+^ (*Green*) on day 10. Cell nuclei were stained with DAPI (*Blue*). Scale Bars: D, 100 μm. E, F & G, 200 μm. *n* = 3 for each group

Following the dual inhibition protocols, we further explored the role of FGF2 signaling in FP induction or patterning. Inactivation of FGF signaling has been reported to disable neural induction from ESCs [21]. It has been argued that FGF2 signaling inhibits neural induction of hESCs and inhibition of FGF2 signaling blocks ESCs entering the epiblast-like intermediate stage [22]. In our differentiation system, after dual restriction with both LDN193189 and SB431542 for the first 24 h, when hESCs have already entered into the NP stage, we found that applying FGF2 for a further 5 days under the dual inhibition protocol resulted in significant down regulation of most neural lineage markers SOX1, FGF5, LMX1A, and MASH1 (Fig. 3a). When FGF2 signaling was continuously inhibited, a severe reduction in cell number was observed. Transient FGF inhibition with PD173074 in the LDN193189 restriction and dual inhibition system resulted in no difference in expression of any of the neural markers. However, when the same 24 h inhibition was performed with PD173074 together with dual inhibition with both LDN193189 and SB431542, a significant up-regulation of SOX1, FGF5, LMX1A and MASH1 was observed (Fig. 3b). Sox1^+^ cells increased to 32.28 ± 4.89 % after the transient inhibition (Fig. 3c), suggesting that transient FGF2 inhibition slows the progression of neural differentiation and sustains the NP state. After dual inhibition, the cells morphologically formed networked structures when switched to differentiation medium with or without transient PD173074 treatment (Fig. 3d). After 5 days, Nestin^+^ and Tuj1^+^ (neuron-specific class III beta-tubulin) cells were found to be co-localized within the colonies formed (Fig. 3e). When sub-cultured, columnar neural rosettes were formed on day 10 (Fig. 3f) with the cells being SOX2^+^ and Tuj1^+^ (Fig. 3g).

### Characterization of FP cell derived dopaminergic neurons in vitro

**Fig. 4:**
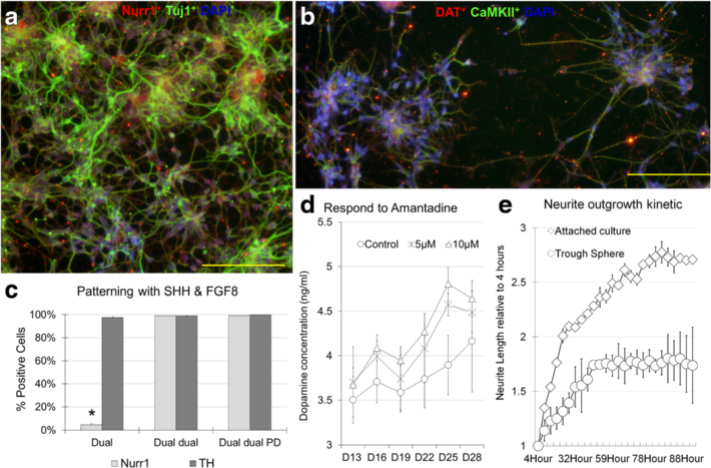
After dual restriction and dual inhibition, cells were subsequently patterned with Shh and FGF8. Cells were Tuj1^+^ (**a** *Green*) and Nurr1^+^ (**a** *Red*), CaMKII^+^ (**b** *Green*) and DAT^+^ (**b** *Red*) after 10 days of patterning. Cell nuclei were stained with DAPI (*Blue*). **c** After 10 days of patterning, for cells subjected to dual lineage restriction followed by culture in N2 supplemented medium, 4.57 ± 0.9 % of the cells were Nurr1^+^, as compared to more than 98 % of Nurr1^+^ cells with continuous LDN193189 treatment or dual inhibition. **d** Dopamine release was found to increase continuously after patterning for up to 28 days. When 5 and 10 μM of amantadine were added to the culture medium, higher dopamine secretion levels were observed and a plateau was reached on day 25 of patterning. **e** Compared with neurons generated from neurosphere, neurons generated from dual restriction and dual inhibition from hESCs single cells displayed a significantly higher neurite outgrowth rate over 90 h culture. Scale Bar: A, B, 100 μm. *n* = 3 for each group

After differentiation with the optimized dual restriction and dual inhibition protocol, cells were subjected to patterning with Shh and FGF8. Immunohistochemical staining for neuronal structural markers and dopaminergic neuron markers was performed after 10 days of patterning, and we observed that the cells were Tuj1^+^ and Nurr1^+^ (Fig. 4a). In addition, we also characterized the electrophysiological properties and dopamine release profiles of these cells as CaMKII^+^ and DAT^+^ (Fig. 4b). Flow cytometry analysis after 10 days of patterning showed that differentiated cells derived from dual restriction and LDN193189 inhibition, dual restriction and dual inhibition, where the majority of the population are SOX1^−^, were more than 98 % Nurr1^+^ and TH^+^. For the dual inhibition and normal induction protocols, where cells were SOX1^+^, only 4.57 ± 0.9 % of the cells were Nurr1^+^ (Fig. 4c). In vitro dopamine release from the cells was also quantified with ELISA after 13 days of patterning. Dopamine release increased from 3.50 ± 0.25 ng/ml to 4.16 ± 0.56 ng/ml after 28 days of patterning. After 5 μM amantadine treatment, dopamine release increased to 3.7 ± 0.39 ng/ml on day13 and 4.48 ± 0.23 ng/ml on day18. After 10 μM treatment, there was no further increase in dopamine release on day13. However, dopamine release increased to 4.64 ± 0.20 ng/ml on day 18 (Fig. 4d). The increase in dopamine release reflects the functional maturation of the dopaminergic neurons over time. When comparing neurite outgrowth after differentiation from FP and neurosphere derived cells, over a 90 h duration, it was observed that the FP derived cells were more active in neurite outgrowth than the neurosphere derived cells. Upon comparing the neurite length at 4 h after seeding, FP derived cells displayed 2.6 folds increment in neurite length, whereas neurosphere derived cells displayed only 1.7 folds increment in neurite length (Fig. 4e).

### FP derived dopaminergic neurons are functional in vitro, survive after transplantation and ameliorate behavioral deficits in PD rats

**Fig. 5:**
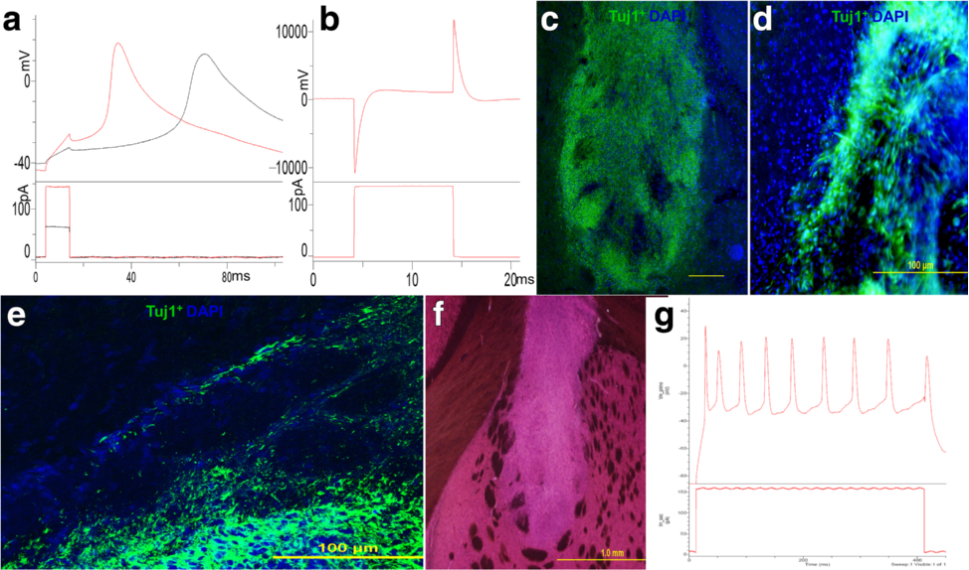
Differentiated dopaminergic cells were subjected to patch clamp analysis. **a** When the cell was exposed to a current of 140pA for 10 ms, membrane potential was found polarized immediately and depolarized after application of the current. When exposed to a current of 70pA for 10 ms, the membrane potential of polarization and depolarization was observed with a delay. **b** Upon exposure to a current of 140pA for 10 ms, depolarization of cell membrane induced the opening of Na_V_ channels, which in turn resulted in an influx of Na^+^ into the cells. Subsequently, opening of K_V_ channels resulted in an efflux of K^+^ out of the cells. **c** After transplanting the cells into hemi-parkinsonian rats, a large number of cells were found to have survived in the host with positive staining for Tuj1 (neuron-specific class III beta-tubulin) at 4 weeks post-surgery. **d** After 6 weeks following transplantation, integration was clearly observable with the transplanted cells interspersed with the host cells. **e** Confocal microscopy showed that some cells have migrated across the host brain nucleus. **f** H&E staining of the host did not show any obvious teratoma formation. **g** Over 8 weeks, when tissues were extracted for electrophysiological studies of grafted cells, action potentials were induced when the cells were exposed to a current of 150 pA for 400 ms. Scale bars: C, D, E, 100 μm. F, 1 mm. Total number of rats transplanted with cells/control culture media is 6 for each group

Before FP derived dopaminergic neurons were transplanted into the PD rat model, whole-cell patch clamp was performed on cultured cells after 20 days of differentiation to investigate their electrophysiological functions. When the cell was exposed to a current of 140pA for 10 ms, membrane potential was found polarized immediately and depolarized after the current was applied. When a current was applied at 70pA for 10 ms, membrane potential at polarization and depolarization was observed with a delay (Fig. 5a). Similarly, upon application of a 140pA current for 10 ms, depolarization of the cell membrane induced the opening of Na_V_ channels, resulting in the influx of Na^+^ into the cells. Subsequently, opening of K_V_ channels resulted in efflux of K^+^ out of the cells (Fig. 5b).

After transplantation of cells into the stratum of the host, tissue samples were collected from animals at week 4, 6, and 8 to assess cell survival and integration with β-III tubulin immunostaining. At 4 weeks, a large number of cells were found to have survived within the host with positive staining for β-III tubulin (Fig. 5c). After 6 weeks, integration was clearly observable with transplanted cell interspersed with host cells (Fig. 5d). Some cells were found to have migrated from the injection site to the surrounding host tissue by week 8. Confocal microscopy revealed that some cells have migrated across the host nucleus (Fig. 5e). After H&E staining of the host tissue, no obvious teratoma formation was found at both low and high magnifications (Fig. 5f). Over 8 weeks, clear action potentials were recorded from grafted cells within the host. When tissues were extracted for electrophysiological studies of the grafted cells, action potentials were induced when the cells were exposed to a current of 150 pA for 400 ms (Fig. 5g). The appearance of the action potentials indicates that the cells already had the key functional property of neurons.

**Fig. 6:**
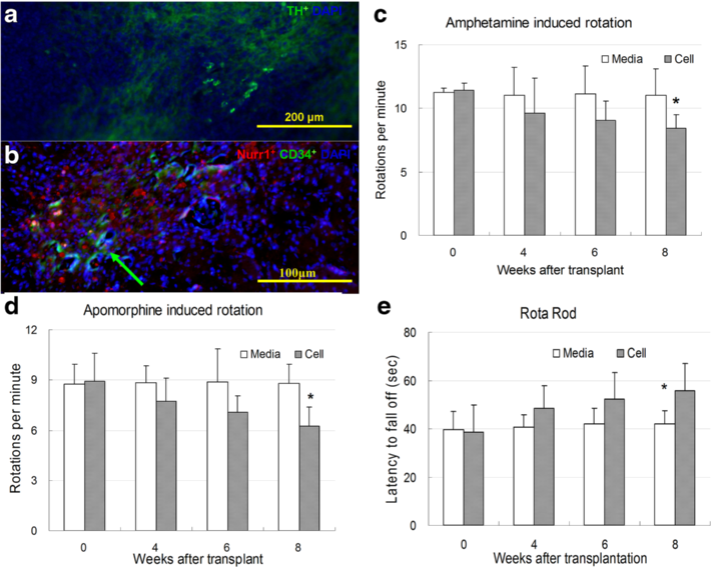
**a** In host rat tissue harvests at 8 weeks post-transplantation, there was positive staining with a human-specific anti-tyrosine hydroxylase (*Red*) antibody utilized as a human-specific and dopaminergic cell-specific marker. **b** Grafted cell and host vascular congruency were clearly observed after CD31 (*Green*) staining, which was both rat-specific and an endothelial cell specific marker. **c** At 8 weeks post-transplantation, amphetamine induced ipslateral rotation in the treatment group was significantly reduced, as compared to the control sham-transplanted group. **d** There was a significant reduction in apomorphine induced contra-lateral rotations compared to the control at 8 weeks post-transplantation. **e** Animals were studied with rota rod and a significant increase in latency to fall was observed in groups of transplanted animals compared to the sham-transplanted controls. Scale bars: A, 200 μm. B, 100 μm

In host rat tissues harvested at 8 weeks after transplantation, there was positive staining with a human-specific anti-tyrosine hydroxylase antibody utilized as a human-specific and dopaminergic cell-specific marker (Fig. 6a). To further investigate graft-host interaction, a rat specific vascular marker CD31 was targeted together with counterstaining that employed a human specific Nurr1 antibody in host tissue (Fig. 6b). At 8 weeks after transplantation, amphetamine induced ipslateral rotation in the treatment group displayed a significant reduction compared to the control sham-transplanted group (Fig. 6c). Similar to amphetamine induced ipslateral rotations, improvements in apomorphine induced contra-lateral rotations also displayed a significant reduction compared to the control at week 8 (Fig. 6d). To assess motor activities, animals were studied with rota rod and a significant increase in latency to fall was observed in groups of animals transplanted with the cells, as compared to the sham-transplanted controls (Fig. 6e).

## Discussion

Within the FP, there are two distinct populations of cells located in the lateral and medial regions of the FP. It is generally agreed that all FP cells express netrin1, FOXA2 and Shh. However, there is controversy over the origin of FP cells during early embryonic development, particularly the role of notochord in FP induction [23]. Inhibition studies revealed that FP differentiation is impeded along the entire AP axis in mice and chick upon pharmacological blockade of Shh (which is mainly expressed in the notochord) [24, 25]. Gain of function studies demonstrated that high concentrations of Shh induced formation of FP tissue/cells from neural explants and in vitro hESCs culture [8, 26]. Contrarily, loss of function studies in zebra fish revealed unaffected ventral midline differentiation with certain effects on lateral FP induction [27]. Additionally, the finding that surgical ablation of notochord resulted in lack of FP formation was challenged by Nicole and colleagues, who postulated that the absence of FP in notochord ablation experiments was due to removal of FP precursors of nodal origin rather than termination of Shh secretion [28].

It has been shown that Shh is required for lateral FP cell induction, but not for medial FP cells [29]. Medial FP cells are still formed in the absence of a differentiated notochord, thus indicating that Shh signaling originating from the neural tube is not a prerequisite for MFP induction [30, 31]. Additionally, medial FP cells failed to develop in the case of mutations affecting the nodal-related molecule of the TGF-β family despite high levels of Shh expression [32]. It was also reported that signaling from the notochord or medial FP cells can induce lateral FP cells [33]. These data suggest that with differentiating FP cells within in vitro culture, the induction of medial FP cells will also increase the population of lateral FP cells. In this study, it was observed that FP cells, both medial and lateral, can be induced without extrinsic Shh treatment, thus supporting the hypothesis that the developmental fate of FP cells are pre-determined before being exposed to patterning signals from the notochord. It is well established that dorsalization is mediated by BMP and TGF-β signaling, which originate from the epidermis and roof plate [34, 35]. Contrarily, antagonists of the BMP and TGF-β family are known to be secreted from the organizer to protect surrounding cells from dorsalization [36]. BMP4 inhibition from the organizer is required to induce neural differentiation of epiblast cells. However, synchronized TGF-β inhibition stabilizes and accelerates neural induction towards FP lineage fates.

We found that dual restriction under defined culture conditions committed the lineage fate of hESCs to neuroectoderm in only 24 h. Further culture under dual inhibition sustained neural differentiation in favor of the FP lineage fate. Additionally, upon addition of FGF2, the majority of the anterior neural lineage markers were down-regulated, suggesting that after neural induction of hESCs, in the early phase of neurogenesis, high levels of FGF2 inhibits the anterior neural fate by up-regulating pluripotency markers OCT4 and NANOG expression. It has been shown that FGF2 acts as a morphogen for the anteroposterior axis of the CNS and that FGF2 induces gastrula ectoderm cells to express posterior neural markers in Xenopus [37]. Other studies have shown that FGF2 caudalizes intermediate neural tissue, inducing spinal cord formation from anterior tissue [38]. In our in vitro culture system, endogenous FGF2 could act in two ways simultaneously, ensuring cell survival and inducing differentiation into caudal neural tissues. In this study, when FGF2 inhibitor is added to the culture for only 24 h, significant FP induction was observed and maintained. Our data thus indicates that neural conversion occurs in the presence of weak FGF signaling combined with BMP inhibition, and that pre-gastrulation FGF signaling is required in the ectoderm for the emergence of neural lineage fates [39]. Hence, inhibition of FGF signaling should be in a temporal manner to ensure cell survival during neural induction. In combination with ventralization factors that block BMP and TGF-β signaling, SOX1^−^ FP cells were induced after 5 days and these cells can be further induced into dopaminergic neurons efficiently. In contrast, despite the expression of other FP genes, dopaminergic cells cannot be efficiently induced in culture when cells are still expressing SOX1 and Nurr1.

Following the pioneering study of Kriks et al. [7], a number of other studies have also attempted to utilize a similar floor plate-based strategy for the derivation dopaminergic neurons from human pluripotent stem cells [40–43]. Nevertheless, all of these studies [40–43] including that of Kriks et al. [7], carried out floor plate induction through stimulation of the Shh signaling pathway, either through the direct use of recombinant Shh protein itself, or it’s agonist purmorphamine. Our study differs from these previous studies by utilizing a Shh-free approach to floor plate induction, through dual inhibition of the BMP4 (LDN193189) and TGF-β signaling pathways (SB431542).

The results of this study demonstrated that the hESC-derived dopaminergic neurons are functional in vitro, can survive after transplantation and integrate within the host brain. This is thus consistent with a previous study which showed that grafted dopaminergic neurons possess an intrinsic capacity for innervation of the adult striatum [44]. Significant functional improvements were observed in amphetamine induced ipslateral rotations, apomorphine induced contra-lateral rotations and Rota rod motor tests over a duration of 8 weeks post-transplantation, and it is anticipated that the grafted cells would continue to provide functional improvement at later time points, because the long-term survival and health of dopaminergic neuron transplants in Parkinson’s disease patients over several years have been conclusively demonstrated [45]. Nevertheless, it is unclear whether the observed amelioration of behavioral deficits in the rat PD model arise directly from new synaptic connectivity conferred by the transplanted cells, or is instead due to other pro-survival, neuroprotective or trophic effects of paracrine secretions emanating from the transplanted cells. Although the results of this study appear promising, we still need to investigate what affects the survival of grafted cells in host, how the grafted cell integrate with host and how the dopamine release from grafted cells are controlled. By using an optogenetic tool, we could be able to shut down the biological function of the grafted neurons within the host brain and successfully created a temporal lapse of behavioral restoration in animal models. In a previous study [46], Wentz and colleagues developed a wirelessly powered control LED system for delivering light into specific areas of the brain. Perhaps, we could utilize similar instrumentation for future optogenetic studies to dissect the cellular and molecular function of grafted dopaminergic neurons within the rat brain.

## Conclusion

We have demonstrated that when cells were predominantly SOX1^−^ under dual inhibition with both LDN193189 and SB431542, FP cells can be generated efficiently. Following patterning with Shh and FGF8, midbrain dopaminergic neurons can be efficiently derived at a very high yield. Our findings thus provide a convenient approach to FP development and functional dopaminergic neuron derivation.

## Additional files

Additional file 1: Table S1. Sequences of primers utilized for RT-PCR analysis. (DOCX 14 kb) Additional file 2: Table S2. Primary and secondary antibodies utilized for immunohistochemistry. (DOCX 13 kb) Additional file 3: Figure S1. Analysis of neural markers expression after treatment with 1, 2 and 5 μM of LDN193189 for 24 to 72 h. Untreated hESC was utilized as the control. Treatment with 2 μM LDN193189 for 24 h yielded the highest neural marker expression on average, as comparing with other dosages. Although there was marked increase in SOX1 expression after 48 h treatment with 2 μM LDN193189, low cell viability was observed. (TIF 766 kb)
