# Supplementary material for: Efficient derivation of dopaminergic neurons from SOX1− floor plate cells under defined culture conditions
Source: J Biomed Sci. 2016 Mar 8;23:34. doi: 10.1186/s12929-016-0251-6 (PMC4782356; doi:10.1186/s12929-016-0251-6)
Supplement: Additional file 1: Table S1. — Sequences of primers utilized for RT-PCR analysis. (DOCX 14 kb) [file 12929_2016_251_MOESM1_ESM.docx]

**Additional file 1: Table S1**

| Gene | Primer | Sequence |
| --- | --- | --- |
| Oct4 | F | 5-CGTGAAGCTGGAGGAAGAGAAGCTG-3 |
|  | R | 5-AAGGGCCGCAGCTTACACATGTTC-3 |
| Nanog | F | 5-TGATTTGTGGGCCTGAAGAAAA-3 |
|  | R | 5-GAGGCATCTCAGCAGAAGACA-3 |
| Nestin | F | 5-CTCGAGCAGGAAGTGGTAGG-3 |
|  | R | 5-TTGGGACCAGGGACTGTTAG-3 |
| Pax6 | F | 5-AACAGACACAGCCCTCACAAACA-3 |
|  | R | 5-CGGGAACTTGAACTGGAACTGAC-3 |
| Sox1 | F | 5-CTCACTTTCCTCCGCGTTGCTTCC-3 |
|  | R | 5-TGCCCTGGTCTTTGTCCTTCATCC-3 |
| NCAM | F | 5-AGGAGACAGAAACGAAGCCA-3 |
|  | R | 5-GGTGTTGGAAATGCTCTGGT-3 |
| DCX | F | 5-AATCCCAACTGGTCTGTCAAC-3 |
|  | R | 5-GTTTCCCTTCATGACTCGGCA-3 |
| Mash1 | F | 5-GTCGAGTACATCCGCCTG-3 |
|  | R | 5-AGAACCAGTTGGTGAAGTCGA-3 |
| FGF-5 | F | 5- CCCGGATGGCAAAGTCAATGG -3 |
|  | R | 5- GGTGACCTTCATGGTGGG -3 |
| EN1 | F | 5- CGTGGCTTACTCCCCATTTA -3 |
|  | R | 5- TCTCGCTGTCTCTCCCTCTC-3 |
| FOXA2 | F | 5- CCGTTCTCCATCAACAACCT -3 |
|  | R | 5- GGGGTAGTGCATCACCTGTT-3 |
| LMX1A | F | 5- CGCATCGTTTCTTCTCCTCT -3 |
|  | R | 5- CAGACAGACTTGGGGCTCAC -3 |
| SIX3 | F | 5-ACCGGCCTCACTCCCACACA-3 |
|  | R | 5-CGCTCGGTCCAATGGCCTGG-3 |
| GBX2 | F | 5-GTTCCCGCCGTCGCTGATGAT-3 |
|  | R | 5-GCCGGTGTAGACGAAATGGCCG-3 |
| OTX2 | F | 5-ACAAGTGGCCAATTCACTCC-3 |
|  | R | 5-GAGGTGGACAAGGGATCTGA-3 |
| IRX3 | F | 5-GGCTTGCGCCCCGTAGAAATGT-3 |
|  | R | 5-AGGAGCCAGGTCAGGTCCGAAC-3 |
| NTN1 | F | 5-GCATGCAGGTTGCAGTTACA-3 |
|  | R | 5-GCTGCAAGCCCTTCCACTA-3 |
| SOX2 | F | 5-TGGTCCTGCATCATGCTGTAG-3 |
|  | R | 5-AACCAGCGCATGGACAGTTAC-3 |
