# Supplementary material for: Efficient derivation of dopaminergic neurons from SOX1− floor plate cells under defined culture conditions
Source: J Biomed Sci. 2016 Mar 8;23:34. doi: 10.1186/s12929-016-0251-6 (PMC4782356; doi:10.1186/s12929-016-0251-6)
Supplement: Additional file 2: Table S2. — Primary and secondary antibodies utilized for immunohistochemistry. (DOCX 13 kb) [file 12929_2016_251_MOESM2_ESM.docx]

**Additional file 2: Table S2**

| **Primary antibodies**  **Protein target** | **Species** | **Commercial**  **source** | **Dilution**  **factor** |  |
| --- | --- | --- | --- | --- |
| Oct-04 | Rabbit | Santa Cruz | 1:200 |  |
| SSEA4 | Mouse | Santa Cruz | 1:400 |  |
| SOX2 | Rabbit | Santa Cruz | 1:200 |  |
| Doublecortin (DCX) | Rabbit | Santa Cruz | 1:200 |  |
| Nestin | Rabbit | Milllipore | 1:400 |  |
| Pax6 | Mouse | Santa Cruz | 1:200 |  |
| SOX1 | Goat | Santa Cruz | 1:200 |  |
| Camk II | Mouse | Abcam | 1:400 |  |
| GFAP | Rabbit | Santa Cruz | 1:200 |  |
| O4 | Mouse | Millipore | 1:400 |  |
| β-III tubulin (Tuj1) | Mouse | Santa Cruz | 1:200 |  |
| Nurr1 | Rabbit | Millipore | 1:200 |  |
| Sodium Channel (Pan) | Mouse | Sigma Aldrich | 1:200 |  |
| Thyrosine hydroxylase | Mouse | Millipore | 1:400 |  |
| DAT | Rabbit | Santa Cruz | 1:200 |  |
|  |  |  |  |  |
| **Secondary antibodies**  **Protein target** | **Species** | **Commercial**  **source** | **Dilution**  **factor** | **Fluorophore** |
| Mouse IgG | Goat | Life technologies | 1:200 | Alexa Fluor 488 |
| Rabbit IgG | Goat | Life technologies | 1:200 | Alexa Fluor 594 |
| Rabbit IgG | Goat | Life technologies | 1:200 | PE-Cy5.5 |
| Mouse | Rabbit | Life technologies | 1:200 | FITC |
| Goat IgG | Donkey | Santa Cruz | 1:200 | Texas red |
| Goat IgG | Donkey | Santa Cruz | 1:200 | FITC |
