# Supplementary figures and images for: Efficient derivation of dopaminergic neurons from SOX1− floor plate cells under defined culture conditions
Source: J Biomed Sci. 2016 Mar 8;23:34. doi: 10.1186/s12929-016-0251-6 (PMC4782356; doi:10.1186/s12929-016-0251-6)

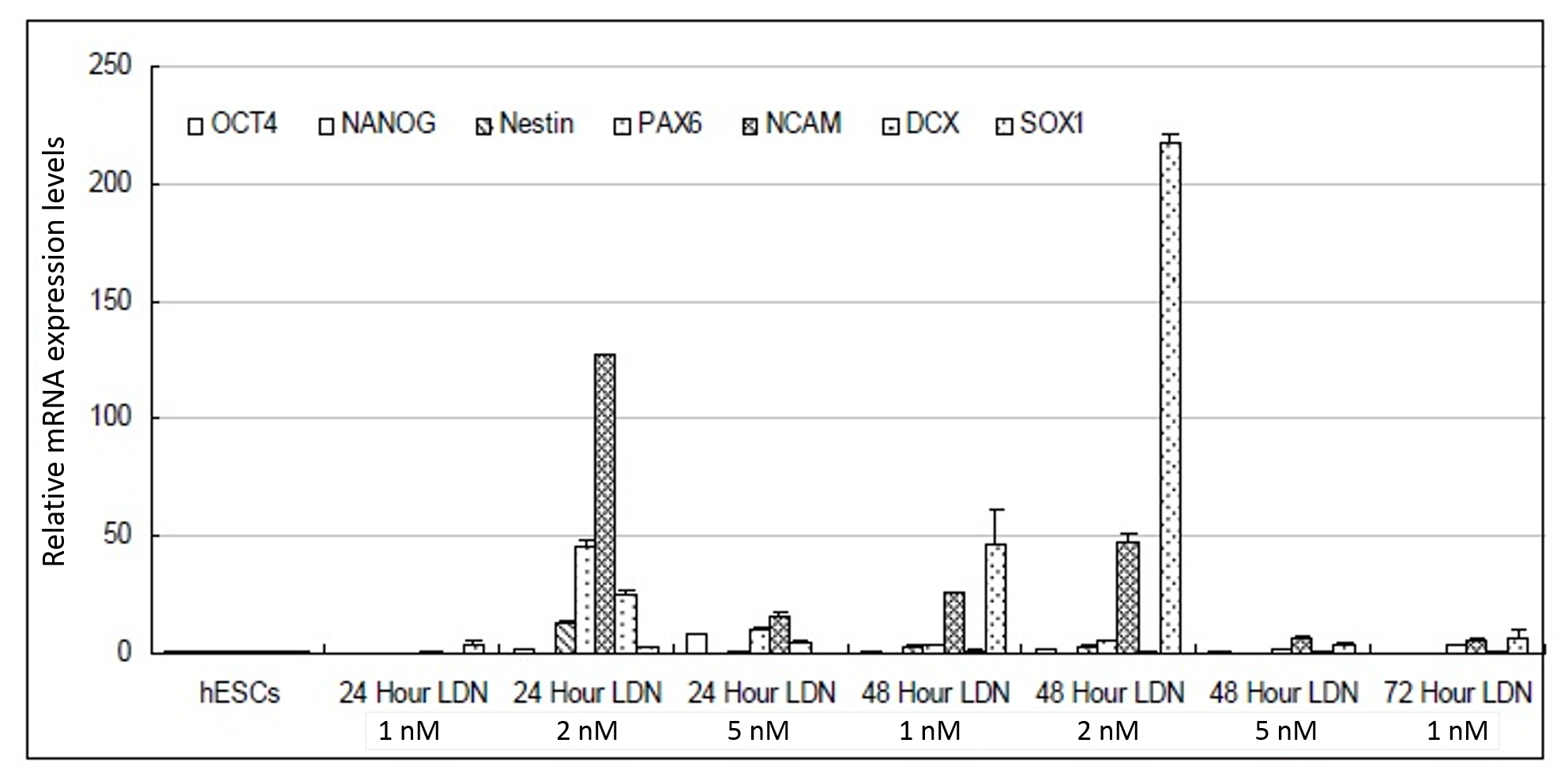

Supplement: Additional file 3: Figure S1. — Analysis of neural markers expression after treatment with 1, 2 and 5 μM of LDN193189 for 24 to 72 h. Untreated hESC was utilized as the control. Treatment with 2 μM LDN193189 for 24 h yielded the highest neural marker expression on average, as comparing with other dosages. Although there was marked increase in SOX1 expression after 48 h treatment with 2 μM LDN193189, low cell viability was observed. (TIF 766 kb) [file 12929_2016_251_MOESM3_ESM.tif]
